# Roles of circRNAs in hematological malignancies

**DOI:** 10.1186/s40364-022-00392-2

**Published:** 2022-07-15

**Authors:** Fahua Deng, Chengsi Zhang, Tingting Lu, Ezhong Joshua Liao, Hai Huang, Sixi Wei

**Affiliations:** 1 Department of Clinical Biochemistry, School of Clinical Laboratory Science, Guizhou Medical University, Guiyang, 550004 Guizhou Province China; 2 Center for Clinical Laboratories, The Affiliated Hospital of Guizhou Medical University, Guiyang, 550004 Guizhou Province China; 3 Key Lab of Endemic and Ethnic Diseases of the Ministry of Education of China in Guizhou Medical University, Guiyang, 550004 Guizhou Province China

**Keywords:** circRNAs, Non-coding RNAs, circRNA functions, Hematological malignancies

## Abstract

As one of the leading causes of death, hematologic malignancies are associated with an ever-increasing incidence, and drug resistance and relapse of patients after treatment represent clinical challenges. Therefore, there are pressing demands to uncover biomarkers to indicate the development, progression, and therapeutic targets for hematologic malignancies. Circular RNAs (circRNAs) are covalently closed circular-single-stranded RNAs whose biosynthesis is regulated by various factors and is widely-expressed and evolutionarily conserved in many organisms and expressed in a tissue−/cell-specific manner. Recent reports have indicated that circRNAs plays an essential role in the progression of hematological malignancies. However, circRNAs are difficult to detect with low abundance using conventional techniques. We need to learn more information about their features to develop new detection methods. Herein, we sought to retrospect the current knowledge about the characteristics of circRNAs and summarized research on circRNAs in hematological malignancies to explore a potential direction.

## Introduction

Hematologic malignancies, which represent a highly heterogeneous set of blood, bone marrow, and organ-associated diseases, are among the most common neoplasms, with acute leukemia, chronic leukemia, multiple myeloma, lymphoma, and malignant tumors histiocytosis, myeloproliferative disorders to name a few. Moreover, patients plagued by hematological malignancies present with a higher risk for infections and increased relapse rates. Further adding to the plight, acquired drug resistance associated with hematological malignancies poses a clinical challenge. The hard-done work of our peers has illustrated the aberrant expression of various non-coding RNAs (ncRNAs) in the pathogenesis of hematologic malignancies, with several ncRNAs being implicated in the regulation of gene expression at multiple levels, including transcription, translation, and epigenetic modification, thus exerting a myriad of cancer-promoting or cancer-suppressing functions. Meanwhile, ncRNA-based investigations have further reported the involvement of circRNAs in various pathophysiological processes of blood cells, such as blood cell differentiation, proliferation, and apoptosis of blood cells, in addition to participating in the occurrence, development and prognosis of hematological malignancies [1].

Circular RNAs, which contain a covalently closed single-stranded RNA, pertain to a category of ncRNAs and are widely expressed and evolutionarily conserved in a wide array of organisms. The first report about circRNAs was published by Sanger HL et al. in 1976 [2], and wherein circRNA molecules were regarded as a mistake-splicing by-product. In 1979, Hsu MT et al. [3] documented the same structure of RNA molecules located in the cytoplasm of eukaryotic cells and termed them as circRNAs. Nevertheless, limitations of detection technology rendered circRNAs as “transcriptional noise” with little immediate attention for years. However, in 1993, Capel et al. [4] illustrated that circRNA SRY was more highly expressed relative to linear SRY in the testis of mice and capable of protein translation. Thereafter, a flurry of investigations has followed and revealed that circRNAs play a crucial part in numerous physiological processes. Furthermore, extensive studies in regard to the mechanism of circRNA in various cancers are already underway.

CircRNAs are known to exhibit solid potential as a predictive, diagnostic and prognostic biomarker, especially their detectability in liquid biopsy samples, including plasma, saliva, and urine. Nevertheless, research related to the role of circRNAs in hematological malignancies is in its infancy and still has a long way to go. In retrospect, the current study summarizes the current knowledge on the biogenesis, regulation, and function of circRNAs and their clinical potential as biomarkers, therapeutic targets, and collaborative drug targets in hematological tumors.

### The biosynthesis of circRNAs

The biosynthesis of circRNAs is inherently dissimilar from the production of mRNAs. Nigro et al. [5] previously documented a disordered RNA transcript, which possessed similar co-splicing sites of DCC (deleted in colorectal cancer) while also containing exons sequences and structures highly-different from DCC. Moreover, the latter research suggested that the by-product might perform a potential function in biological evolution but failed to carry out a further in-depth investigation.

**Fig. 1:**
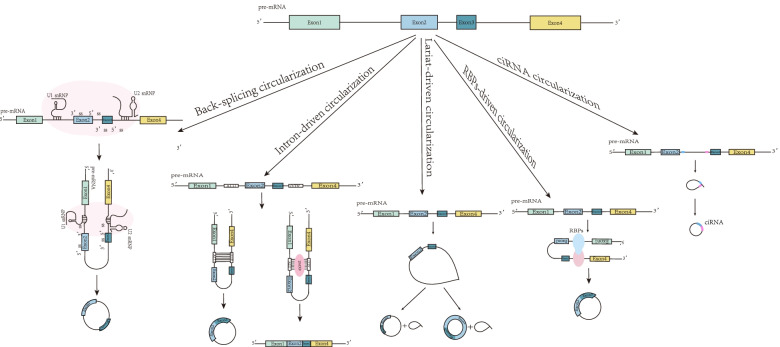
Biosynthesis of circRNAs. CircRNAs can be directly generated by back-splicing; Intron-driven circularization can be inhibited by RNA helicase DHX9; Lariat-driven circularization generates a lariat structure. Spliceosome can splice to generate circRNAs and lariat-introns RNAs. Lariat-introns RNAs can be degraded, but some lariat-introns RNAs can escape degradation to form ciRNAs

The advent of enhanced technologies, like circRNA microarray and high-through sequencing, has further revealed three biosyntheses mechanisms [6] of circRNAs (Fig. 1): first, back-splicing circularization: Back-splicing is a phenomenon seen in a vast majority of eukaryotes, wherein small nuclear ribonucleoproteins (snRNPs) catalyze the pre-mRNA 5′ donor site to attack 3′ receptor site. Subsequently, a 3′-5′ phosphodiester bond formation ensues, which can be used to connect circRNAs covalently. Second, intron-driven circularization: Circularizable exons are flanked by long introns rich in ALU components to enhance complementary pairing and form repeatedly reverse-complementary secondary structures. Subsequently, the upstream and downstream exons covalently form circRNAs. Interestingly, the report published by Ashwal-Fluss R et al. [7, 8] indicated that flanking introns participated in circRNA generation and the quantity of ALUs exerted a crucial role in circularization efficiency. Third, lariat-driven circularization: The exon splicing donor and receptor combine as a “lariat intermediate,” followed by spliceosome removal of redundant introns. It is worth noting that the accelerated generation of lariat RNAs can be mediated by templates and lariat RNAs, such that lariat RNAs are also capable of generating circRNAs and linear branched-chain RNAs via the debranching enzyme [9].

A variety of splicing factors regulate the process of circRNA generation, including cis−/trans-acting splicing regulatory elements [6], which are combined with splicing factors to enhance or inhibit the expression of circRNAs. However, it is worth noting that splicing factors in circRNA generation may confer a different role in linear RNAs. Besides, circRNAs possess the ability to regulate circularization by binding to RNA-binding proteins (RBPs). For instance, circCAMSAP1 in colorectal cancer can directly bind with Epithelial-splicing regulatory protein 1 (ESRP1) to promote circularization of circCAMSAP1 [10]; and QKI can induce normal linear transcripts de novo synthesis and generate circRNAs [11]; whereas, DHX9 can inhibit the formation of ALU’s complementary structures by binding to ALU elements to prevent circRNA production [12].

Moreover, some lariat-introns RNAs can escape from degradation to form circular intronic RNAs (ciRNA). A consensus motif is containing a 7 nt GU-rich element near the 5′ splice site and an 11 nt C-rich element close to the branchpoint in ciRNA-producing introns can help intron lariat escape from the debranching enzyme [13].

Additionally, circRNA biosynthesis can also be affected by some specific physiological conditions, such as immune response [14]. RNase L is activated to rapidly degrade circRNAs with virus infection, which leads to activation of the PRK pathway and leading to a cascade of activation of innate immunity.

### The characteristics of circRNA

#### CircRNAs are widely-expressed and conserved

CircRNAs are expressed in a wide array of organisms, like animals, plants, bacteria, archaea, etc. [4, 15, 16], and sequencing of circRNAs in different organisms has revealed that circRNAs are more conservative than linear RNAs. In addition, higher expression of circRNAs is associated with enhanced conservative properties. For example, circRNAs TTBK2, Ttbk2 and Asator are highly expressed in drosophila’s brains were also found in mammalian brains and exhibit identical sequences [16, 17]. Meanwhile, high conservatism of circRNAs is indicated in exons as reflected in flanking intron sequences of circularization exons, exact back-splicing sites, and expression patterns.

#### CircRNAs are expressed with exquisite tissue/cell-specificity

CircRNAs are highly expressed in mammalian brain tissues. Like circMfsd6 and circZfp609, some circRNAs present with significantly higher expression relative to their homologous linear RNAs [17]. Further investigation by Rybak-Wolf et al. has revealed that circRNAs are primarily enriched in axons, dendrites, and synapses [17, 18]. The types and expressions of circRNAs are transformed in diverse differentiation stages to exert crucial functions in neuronal differentiation and maintenance. Meanwhile, the study carried out by You et al. has reported that circRNAs are specifically distributed in different subcellular organelles and developmental stages of cells with qRT-PCR and RNA-seq [18].

#### High stability is a common feature of circRNAs

**Fig. 2:**
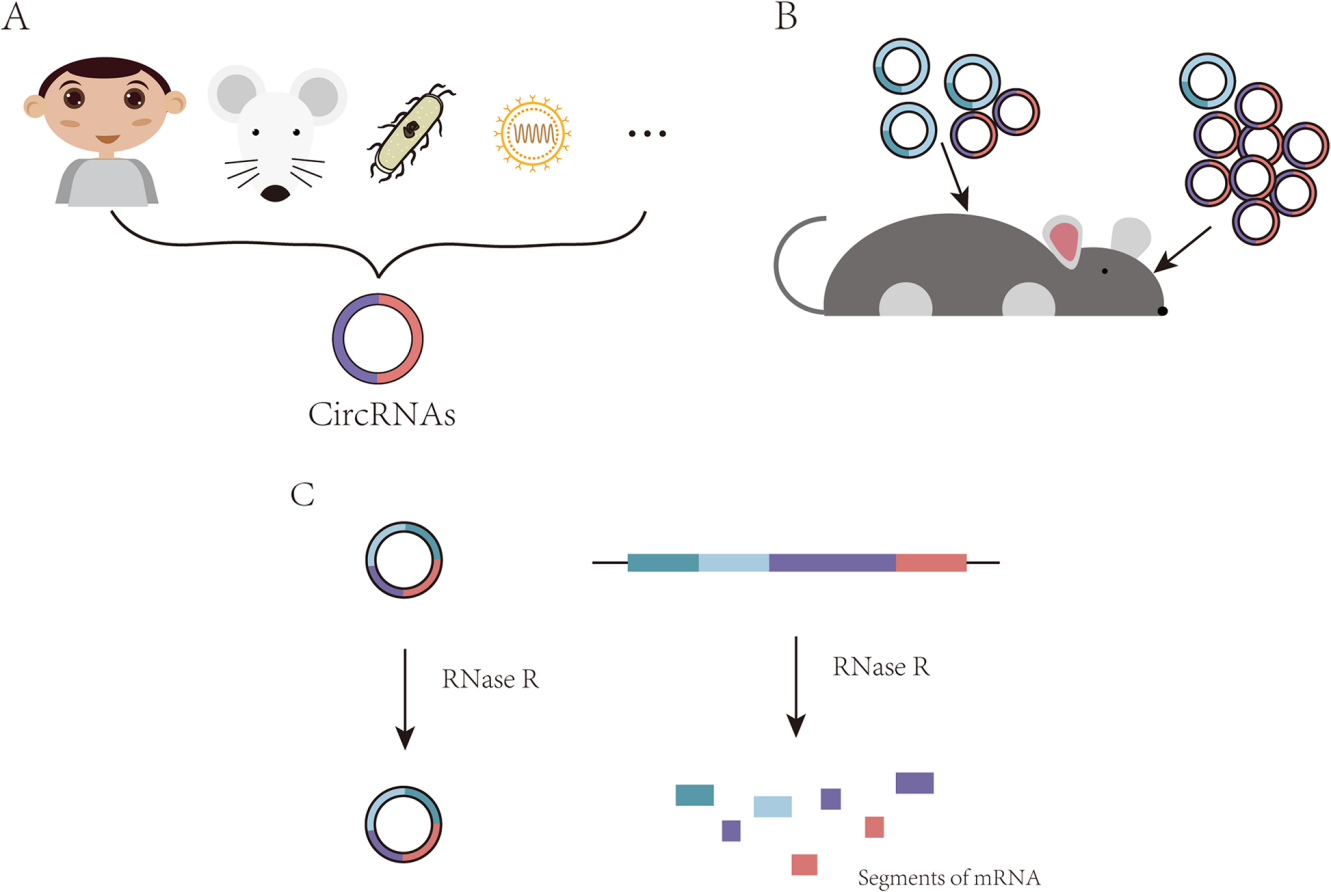
Characteristics of circRNAs. **A** CircRNAs are widely expressed in various organisms; **B** CircRNAs are expressed in a tissue-specific manner; C. CircRNAs with high stability can resist degradation from the exonuclease

With advantages such as cyclic structures and lack of free ends, circRNAs are hardly recognized by the exonucleases to resist degradation from the exonuclease exposure. CircRNAs are also known to exhibit stability following treatment with actinomycin D treatment for 48 hours [16]. In addition, circRNAs have a longer half-life, approximately 2.5 times longer than their counterparts linear RNAs [19, 20]. The work of Dongming Liang et al. [21] has previously indicated that damaging RNA splicing mechanism can promote circRNAs to switch to a non-promoter structurally dependent transcription mechanism. These aforementioned evidence indicated that circRNAs maintained high stability and the potential for long-term regulation of cell behaviors (Fig. 2).

### The functions of circRNAs

#### CircRNAs regulate transcription

Despite the differences in the biosynthesis of circRNAs and linear RNAs, both are catalyzed by conventional splicing signals and canonical splicing system so that circRNAs can serve as an “mRNA trap” to interfere with linear counterparts mRNA competing with the binding site of the spliceosome [22]. Moreover, flanking introns on circularization exons on pre-mRNA can also bind with the spliceosome and influence the fate of mRNA biosynthesis [7].

Moreover, the study carried out by Xiang Li et al. has illustrated that ciRNA ci-ankrd52 maintains R-loop structure to facilitate transcriptional elongation across its producing locus [23].

#### CircRNAs act as miRNA sponges

After uncovering the presence of miRNA-binding sites, further experimentation has revealed that circRNAs function as “miRNA sponges” and indirectly regulate the expression of mRNAs [24–26]. For instance, circRNA MAT2B [25] sponged miR-338-3p to promote glycolysis and malignancy in hepatocellular carcinoma (HCC), while circPAN3 [26] mediated drug resistance in acute myeloid leukemia through binding with miR-153-5p/miR-183-5p.

CircRNAs further possess multiple miRNA response elements of different miRNAs, such as circHIPK3 [27], while there is also evidence to suggest that multiple circRNAs can regulate the same miRNA simultaneously. Subsequently, the miRNAs initially targeted mRNA will bind with circRNAs, and form a circRNA-miRNA-mRNA competing endogenous RNAs (ceRNA) network to regulate mRNA expression. Except for sponges, circRNAs can act as reservoirs to enrich miRNAs and enhance the effect. As an example of the latter, circ-DOCK5 reserve miR-627-3p to repress metastasis in squamous cell carcinoma [28]. Moreover, circRNAs are capable of regulating regulated miRNA biosynthesis via changing the intracellular localization of DICER [29].

#### CircRNAs interact with proteins

Most circRNAs, which contain multiple RBPs binding sites, were located in the cytoplasm. Herein, circRNAs could work as “decoys” or “scaffolds” to bind to RBPs, isolate RBPs from their target molecules, mediate the intracellular localization of RBPs [30], and thereby regulate RBP biosynthesis, transport, and influence subcellular biological processes [1]. CircRNAs might directly or indirectly crosstalk by transcription, for example, circRHOT1 [31] can recruit TIP60 to NR2F6 promoter to activate NR2F6 to enhance HCC progression. Meanwhile, circFoxo3 can interact with MDM2 and p53 to augment MDM2-induced p53 degradation [32].

#### Exosomal circRNAs take part in cellular communication

Exosomes are extracellular vesicles with a size range of ~ 40 to 160 nm (average ~ 100 nm) in diameter [33], and serve as an intercellular transit system with pleiotropic functions. In 2015, the study performed by Li et al. [34] has illustrated that circRNAs are enriched and stable in exosomes by RNA-seq. In addition, the previously mentioned study also documented more than 1000 circRNAs in human serum exosomes.

Owing to the stability of circRNAs, exosomal circRNAs can be carried to participate in cellular information transmission and disease progression. For example, serum exosomal circRNA-104484 and circRNA-104670 are significantly enhanced in sepsis [35], and possess the potential as diagnostic markers in sepsis. Exosomal circRNAs can also regulate cancer development; for instance, exosomal circRNA-100338 is capable of regulating angiogenesis and metastasis of HCC, while serum exosomal circRNA-100338 can predict lung metastasis of HCC patients following curative hepatectomy [36]. Similarly, exosomal circRNA-133 of CRC patients can transport normoxic cells to hypoxic cells, regulate the E-cadherin membrane distribution, and promote cancer metastasis via miR-133a/GEF-H1/RhoA axis [37].

#### CircRNAs regulate epigenetics

Epigenetic regulation is relevant to promote the occurrence and development of multiple diseases in the most abundant type of cells. Currently, numerous data have indicated that N6-methyladenosine modification can modulate the expression, distribution and functions of circRNAs [38]. On the other hand, some circRNAs are previously reported to regulate the expression, functions and proteins interaction of m6A, such as circMAP2K4 sponging miR-139-5p can regulate the expression and activity of YTHDF1 [39]. Meanwhile, circNOTCH1 competitively binds with METTL14 to release NOTCH1 mRNA [40].

#### CircRNAs translate proteins

Due to a lack of the cap structure and poly adenylate tail, circRNAs were translated by spliceosome activity-dependent and cap-independent methods. The internal ribosome entry site (IRES) [41], located in the 5′ UTR, serves as the main pathway for circRNA translation. IRES exerts its functions by several mechanisms, including analogous RNA structures interacting with 18S rRNA and binding with IRES trans-acting factor ITAF. Losing the cap structure of circRNAs drives IRES activation and recruits IRES factors to initiate circRNA translation. Simultaneously, activated IRES can enhance circRNA translation efficiency upon undergoing stress, such as heat shock, cancer, and hypoxia. IRES has no advantage in overactivity and number in vivo, and thus circRNAs exhibit a lower translation efficiency than linear RNAs, which usually are ten times as much as circRNAs [42]. Nevertheless, circRNA translation can be enhanced in various ways, like m6A modification [43] in eukaryotic circRNAs to help achieve circRNA translation.

**Fig. 3:**
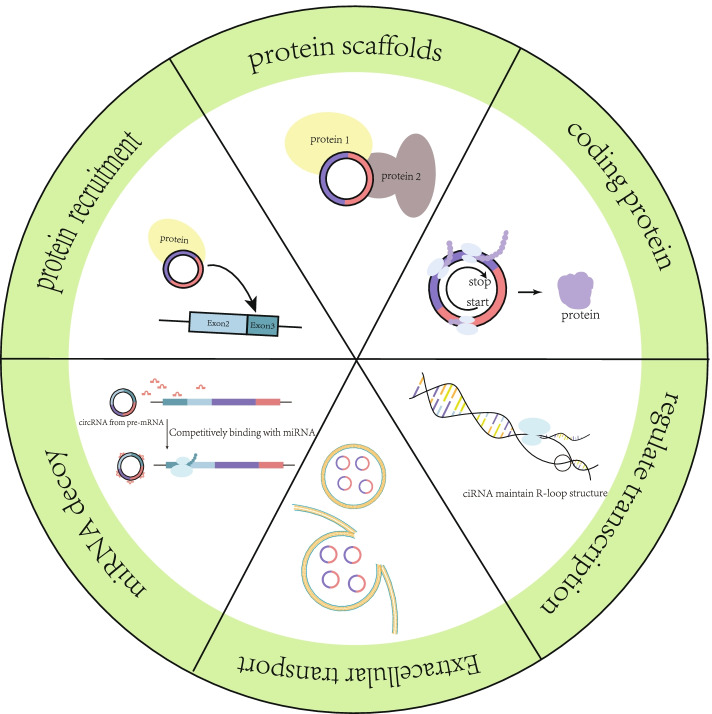
Functions of circRNAs. CircRNAs can maintain R-loop structure to facilitate mRNA transcription and indirectly regulate mRNA transcription by recruiting proteins or acting as ceRNA to compete with mRNA. Besides, circRNAs act as a protein scaffold to mediate protein-protein interactions, protein localization, and protein transport. Moreover, circRNAs code proteins and regulate nearby cells by exosome transport

It was interesting to explore the discrepancy in translated methods between circRNAs and linear RNAs by studying translated products from circRNAs. A novel translation mechanism previously indicated to produce particular proteins: rolling circle translation [20, 44, 45]. Covalently closed circRNAs are translated when the number of nucleotides contained in the open reading frame (except for the stop codon) are an integer multiple of 3, whereas the termination codons did not take participate in translation (Fig. 3).

### CircRNAs in hematological malignancies

More and more evidence has further classified ncRNAs and associated with hematopoiesis and hematological malignancy initiation, including miRNAs, long non-coding RNAs (lncRNAs), and circRNAs.

#### CircRNAs in blood cell differentiation

Hematopoietic stem cell (HSC) differentiation is strictly regulated by multiple factors, including transcription factors, ncRNAs (including circRNAs), and other factors. Accumulating reports have shown that an array of circRNAs are expressed explicitly in the continuously hematopoietic process [46–48]. CircSPI1 can be up-regulated to inhibit myeloid differentiation of acute myeloid leukemia (AML) cells [49]. Moreover, quantitative detecting of circRNAs in various blood cells by Nicolet et al. [47] illustrated the presence of changes in circRNA type and quantity in addition to the maturation and differentiation of HSCs. CircRNAs are similarly up-regulated in mature erythrocytes and platelets, and Nicolet et al. [46] further indicated their association with translation and housekeeping function exertion in mature erythrocytes. However, there is still little evidence on whether circRNAs can translate proteins in red blood cells, questioning their potential as molecular markers to distinguish differentiation stages and requiring further elaboration.

#### CircRNAs act as biomarkers in hematological tumors

CircRNAs are regarded as diagnostic and prognostic biomarkers to indicate the development of hematological tumors and therapeutic responses. In 2012, hundreds of circRNAs were documented in AML patients by circRNA microarray and genome-wide microarray analysis [48]. More and more circRNAs have been attested to serve as potential clinical biomarkers. For instance, circAML1, which was transcribed from AML1, which acts as an oncogene of AML, is up-regulated in the bone marrow and peripheral blood [50], while being significantly down-regulated in bone marrow-derived exosomes of essential thrombocythemia patients. Those studies hinted that circAML1 maybe serve as a biomarker in the hematological system [51]. Moreover, high-throughput analysis and bioinformatics analyses have screened and validated differentially expressed circRNAs in AML, underscoring the ability of circ-0004277 as a potential diagnostic marker and therapeutic target for AML [52].

Accumulating evidence further indicates that circRNAs also contribute to discovering the development of disease. For example, circ-VIM was positively correlated with AML progression, wherein circ-VIM served as a pro-oncogenic circRNA to accelerate the progression of AML [53]. Meanwhile, circ-0000190 can serve as an independent risk factor for risk stratification and the prognosis of MM [54], while circ-0004277 [52] was previously associated with risk status and treatment response in AML. Hsa-circ-100352, hsa-circ-104056, and hsa-circ-102817 are differentially expressed and promote the progression of MDS and are related to the survival and prognosis of MDS [55–57].

On the other hand, up-regulation of circ-ITCH was was negatively associated with ISS in MM patients and positively correlated with progression-free survival (PFS) and overall survival (OS) [58]. Similarly, circ-VIM was negatively associated with OS and leukemia-free survival (LFS), and reported to serve as an independent poor prognostic factor for OS and LFS in AML patients [59].

#### CircRNAs regulate the proliferation, apoptosis, cell cycle, invasion and migration of hematological malignancies

Proliferation misregulation has been indicated as one of the critical factors in hematological malignancies. Recent studies demonstrated the correlation between circRNA expression and cell proliferation in hematological malignancies. For instance, circ-0004136 [60] promoted AML cell proliferation by sponging miR-142, while circ-0001947 [61] up-regulated CREBRF to repress AML cell proliferation by inhibiting hsa-miR-329-5p. In addition, circPOLA2 [62] and circ-0121582 [63] were over-expressed in AML and promoted cell proliferation by targeting their miRNAs. Similarly, circMYBL2 in AML serves as a potential therapeutic target and it regulates the level of FLT3 kinase by interacting with PTBP1 to inhibit proliferation [64].

Avoiding apoptosis represents a crucial factor in promoting the hematological malignancies process. Accumulating studies have indicated that circRNAs can regulate cell apoptosis and cell cycle progression during hematological malignancies. For example, both circCDYL [65] and its targeted gene YAP undergo up-regulation to inhibit apoptosis. Moreover, circRNAs can serve as essential factors for the evasion of the death of cells, as circKEL [66] inhibits the apoptosis of AML via sponging miR-335-5p, and up-regulation of circ-0009910 [67, 68] in CML and AML was associated with inhibition of apoptosis by the miR-34a-5p/ULK1 axis. Knockdown of circPTK2 [69] can augment the expression of miR-330-5p and decrease the targeted mRNA FOXM1 to promote apoptosis.

In addition to regulating apoptosis and proliferation, circRNAs can also exert control over cell cycle progression to influence the progression of hematological malignancies process. CircCBFB [70] was previously associated with the cell cycle to facilitate CLL development. Moreover, circ-0002483 [71] could further accelerate the cell cycle period by sponging miR-758-3p.

Meanwhile, circRNAs have been shown to regulate the invasion and migration of cells and further promote the progression of hematological malignancies, such as overexpressing circ-0069767 decreases the invasion and migration capacities of MM cells by sponging miR-636 to regulate the expression of K-RAS [72], whereas circ-0000142 enhances the invasion of MM cells via the circ-0000142/miR-610/AKT3 axis [73], etc.

#### CircRNAs influence chemo-sensitivity in hematological malignancies

Chemoresistance underlies one of the the key causes of the progression and relapse of hematological malignancies. Some outcomes have suggested that circRNAs can assist in overcoming chemoresistance. For example, circANXA2 [74] was associated with chemoresistance of cytarabine and daunorubicin in AML. Similarly, circPAN3 [26] induced chemoresistance of adriamycin (ADM) via sponging miR-153-5p/miR-183-5p, while bortezomib (BTZ)-circ-0003489 [75] may regulate resistance in MM. Circ-0009910 triggered the activation of autophagy by sponging miR-34a-5p to promote imatinib resistance in chronic myeloid leukemia [68].

Therefore, regulating chemo-sensitivity of hematological malignancies via targeting circRNAs can pave the way for novel clinical therapy and a breakthrough in longer survival time for patients. Table 1

**Table 1:** The role of circRNAs in hematological tumors

Circ based ID / Common Name	Hematological malignancies	Expression pattern	Target miRNA/Target gene	Function	Ref.
circ-0000190	MM	↓	miR-767-5p/MAPK4	Biomarkers of risk stratification and prognosis	[54]
circRPL15	CLL	↑	miR-146b-3p/RAF1	Biomarkers of CLL prognosis	[76]
circ-0004277	AML	↓	WDR37	Biomarkers of risk stratification and therapeutic response	[52]
circDLEU2	AML	↑	miRNA-496/PRKACB	Promoting cell proliferation and inhibiting apoptosis	[77]
circ-0004136	AML	↑	miRNA-142	Promoting cell proliferation and inhibiting apoptosis	[60]
circ-0132266	CLL	↓	miR-337-3p/PML	Promoting cell proliferation and inhibiting apoptosis	[78]
circCBFB	CLL	↑	miR-607/FZD3/ Wnt/b-catenin pathway	Promoting cell proliferation, cell cycle, and inhibiting apoptosis	[70]
circPVT1	ALL	↑	miRNA-let-7 and miR-125/c-Myc/Bcl-2	Promoting cell proliferation, cell cycle and inhibiting apoptosis	[79, 80]
circADD2	ALL	↓	miR-149-5p/AKT2	Promoting apoptosis and inhibiting cell proliferation	[81]
circHIPK3	CML	↑	miR-124	Promoting cell proliferation	[82]
Circ-0009910	CML	↑	miR-34a-5p/ULK1	Promoting cell proliferation and inhibiting apoptosis, increasing chemoresistance of imatinib	[67, 68]
circCDYL	MM	↑	miR-1180/YAP	Promoting cell proliferation and inhibiting apoptosis	[65]
circ-0069767	MM	↑	miR-636/K-RAS	Inhibiting cell proliferation, migration and invasion and promoting cell apoptosis	[72]
circOTUD7A	DLBLL	↑	miR-431-5p/FOXP1	Promoting cell proliferation and migration, inhibiting apoptosis	[83]
circ-0127621	DLBLL	↓	miR-888/APC/Wnt/β-catenin	Inhibiting cell proliferation	[84]

#### F-circRNAs in hematological tumors

**Fig. 4:**
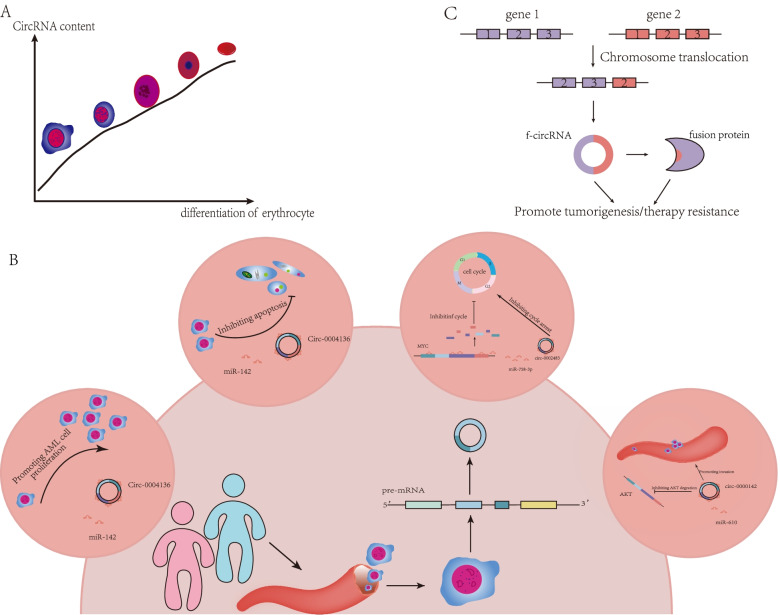
CircRNA in the hematological system. **A** The expression of circRNAs was positively correlated with cell differentiation during erythrocyte differentiation; **B** CircRNAs play functions in hematological tumors; **C** Chromosomal translocation derived f-circRNA, which could translate fusion proteins to promote leukemia

Recently, a novel form of circRNAs--fusion circRNAs (f-circRNAs) was published [57, 88–90], f-circRNAs derived from cancer-associated chromosomal translocation were indicated as one of the critical causes of hematological malignancies. F-circRNAs can function as proto-oncogenes, and up-regulation of f-circRNAs promote the proliferation and clonogenicity, and inhibit apoptosis of leukemia cells, whereas silencing of f-circRNAs is associated with reversal of these phenotypes. A comparison of in vitro and in vivo models for the function of f-circRNAs further highlighted that f-circrNAs can directly lead to leukemia occurrence and promote leukemia formation, maintain the progression, and improve the survival ability of leukemia cells. For instance, circAF4 sponges miR-128-3p to promote cell proliferation and inhibit apoptosis in leukemia [91], whereas circBA9.3 derived from BCR-ABL1 can increase tyrosine kinase activity that promotes resistance against resistance TKI therapy [92]. Moreover, f-circRNA generated from chromosomal translocation can produce many proteins that were entirely different from others, in order to mediate leukemia development, progression, prognostic, and chemoresistance [57, 91] Fig. 4.

### Detecting circRNAs

Accumulating studies have shown that circRNAs only exhibit 1/10000–1/100 of than the expression of their linear counterpart RNAs [93], and because of the same base sequence with counterpart linear RNAs, circRNAs are harder to isolate from total RNA. Accordingly, RNase R is commonly employed to digest the linear RNAs for enriching circRNAs, but it is worth noting that circRNAs can also be digested by long-term RNase R treatment and treated circRNAs should be distinguished from the secondary structure of linear RNAs. For further investigation of circRNAs, high-throughput RNA sequencing can be adopted to detect the differential expression of circRNAs, and bioinformatics (like STAR, circBase) allow the analyses of the functions of passway and the interaction of proteins, etc. Various methods were administrated in the circRNA study. Traditionally used methods in circRNA study are listed as follow [15, 24, 25, 48, 54, 94–96] (Table 2, 3 and 4).

**Table 2:** circRNA influence chemoresistance of hematological malignancies

Circ based ID / Common Name	Hematological malignancies	Expression pattern	Target miRNA/Target gene	Drug resistance	Ref.
circ-0000190	CML	↑	miR-34a-5p/ULK1	Increasing chemoresistance of imatinib	[67, 68]
CircANXA2	AML	↑	miR-23a-5p/ miR-503-3p	Increasing chemoresistance of cytarabine and daunorubicin	[74]
CircPAN3	AML	↑	miR-153-5p/miR-183-5p-XIAP axis/AMPK/mTOR signaling	Inducing chemoresistance of ADM	[26]
circ-0003489	MM	↑	miR-874-3p/HDAC1 axis	Inducing chemoresistance of BTZ	[75]
CircNPM1	AML	↑	miR-345-5p/FZD5 pathway	Increasing Adriamycin resistance	[85]
Circ-ITCH	MM	↓	miR-615-3p/PRKCD axis	Increasing bortezomib sensitivity	[86]
circ-0080145	CML	↑	miR-326/PPFIA1 axis	Increasesing imatinib resistance	[87]

**Table 3:** Methods of circRNA detection

Method	Principle	Purpose	Ref.
Bioinformatics	The online database, such as circBase, Circular RNA Interactome, miRBase, DIANA TOOLS, and Cytoscape	Predicting circRNA interactive network and molecular mechanism	[1, 52]
qRT-PCR	Primers were designed for the back-splicing junction to detect circRNA expression quantitatively	Detecting circRNA expression	[19]
ddPCR	Primers were designed for the back-splicing junction to detect circRNA expression quantitatively	Detecting circRNA expression	[97]
RNase R treatment	RNase R is a magnesium-dependent 3′ → 5′ exoribonuclease, owning that circRNA or lariat lack this structure so that they can resist its digestion	Verifying the structure of circRNA	[1]
Northern blotting	Probes were designed for the back-splicing junction to detect circRNA expression quantitatively	Detecting circRNA expression	[98]
FISH	Fluorescent probes were designed for circRNA and the miRNA binding sites	Analyzing co-location of circRNA and miRNA by in situ imaging	[98]
RCA reaction	CircRNA hybridizes with miRNA to form complex cmRRIs, which trigger RCA reactions. Probes are designed for rolling ring amplification products to detect fluorescence intensity	Detecting circRNA expression and judging the affinity of circRNA to miRNA	[99, 100]
RNA sequencing	The sequence of circRNA was analyzed by high-throughput sequencing	Studying genome-wide differences of circRNAs	[19, 52, 97, 98]
Microarray	CircRNA microarray, which is not affected by RNA abundance, can accurately detect the expression of circRNA in samples by using the double guarantee of a specific splicing site probe and exonuclase pretreatment	Microarray allows fastly, primarily, and more sensitively screen circRNA.	[101]
CRISPR-Cas13 system	CRISPR-RfxCas13d can effectively discriminate circRNAs from mRNAs by using guide RNAs targeting sequences spanning back-splicing junction sites featured in RNA circles	CRISPR-RfxCas13d is a useful tool for the discovery and functional study of circRNAs at both individual and large-scale levels	[96]

**Table 4:** Database of circRNAs

Database	Function	Webpage	Ref.
circBase	Search information of circRNAs sequence	http://www.circbase.org/	[102]
CIRCpedia v2	Search, browse and download circRNAs with expression characteristics/features in various cell types/tissues, including disease samples	http://yang-laboratory.com/circpedia/	[103]
ENCORI	An open-source platform for studying the miRNA-ncRNA, miRNA-mRNA, ncRNA-RNA, RNA-RNA, RBP-ncRNA and RBP-mRNA interactions from CLIP-seq, degradome-seq and RNA-RNA interactome data.	https://starbase.sysu.edu.cn/index.php	[104]
IRESite	Presents information about the experimentally studied IRES (Internal Ribosome Entry Site) segments.	http://www.iresite.org/	[105]
Circular RNA Interactome	Searches circRNAs name, genomic position and best-matching transcripts, RBPs binding site, and information on miRNAs targeting. Designs divergent primers and siRNAs for circRNAs.	https://circinteractome.nia.nih.gov/	[106]
TSCD (Tissue-specific circRNA Database)	Provide a global view of tissue-specific circRNA in the main tissues of humans and mice.	http://gb.whu.edu.cn/TSCD/	[107]
MiOncoCirc	An extensive clinical, cancer-centric resource of circRNAs，it constructed fromclinical cancer samples (2000+) with a plethora of disease sites	https://mioncocirc.github.io/	[108]
TRCirc	Provide transcription factors binding sites (TFBSs) and other correlation information, such as methylation level, H3K27ac signals, super-enhancers and expression of circRNAs.	http://www.licpathway.net/TRCirc/view/index	[109]

### Summary

Herein, the current study revealed the function and clinical significance of circRNAs, and partly recognized their roles in cancer. The biosynthesis of circRNAs is regulated by a plethora of factors, which explains the involvement of circRNAs in various physiological processes in an organism. Moreover, the interaction between circRNAs and various molecules serves as a mean for participation in transcription, gene expression, protein interaction, and other mechanisms.

In particular, existing evidence indicates that circRNAs bears great responsibility for hematopoietic stem cell differentiation and development. CircRNAs are further widely and specifically expressed in blood cells, and exhibit stability in mature erythrocytes. Recent investigations have also shown that circRNAs are differentially expressed in multiple hematologic tumors, and accordingly correlated with the occurrence, development, and prognosis of multiple hematologic tumors. Consequently, it would be plausible to suggest that circRNAs possess the ability to serve as novel biomarkers of hematological malignancies.

Furthermore, up-and-coming researches have indicated that circRNAs exert their functions by accumulating high expression in non-nuclear blood cells. Existing studies have expounded on the ability of circRNAs to promote or inhibit the proliferation, migration and invasion, cell cycle, and apoptosis in hematological malignancies. Meanwhile, circRNA-mediated chemoresistance and exosomal circRNA-mediated tumor microenvironment regulation of adjacent cells or tissues also represent potential directions for hematological malignancies treatment. Moreover, circRNAs, which are differently expressed or newly discovered, can also act as appealing biomarkers for the understanding of the development of hematological diseases. Overall, the emergence of f-circRNAs has opened an innovative door for hematological tumors’ diagnosis and therapy.

CircRNAs also exhibit an excellent promising clinical translation in hematological tumors, however, several biological questions and clinical challenges need to be addressed before their translational potential can be realized. For instance, most of the current studies are based on the available approaches for detection and aimed at the function of the “miRNA sponge,” and the precise mechanisms of circRNAs in hematological malignancies remain elusive. Given that the studies focusing on circRNAs have been hardly abundant, future studies should focus on enriching circRNAs, developing sensitive techniques, etc.

Overall, circRNAs represent potential therapeutic targets that exert their crucial influence on the occurrence, development, prognostic, and treatment of hematological tumors, but many of them are yet to be unveiled. Hopefully, more mechanisms of circRNAs could be clarified shortly, and research could be further expanded.

## Data Availability

Not applicable.

## Abbreviations

ADM: Adriamycin
AML: Acute myelocytic leukemia
BTZ: Bortezomib
ceRNA net: Competing endogenous RNAs net
circRNA: Circular RNA
CLL: Chronic lymphocytic leukemia
DCC: Deleted in colorectal cancer
ddPCR: Droplet Digital PCR
DHX9: DEAH-box helicase 9
ESRP1: Epithelial-splicing regulatory protein 1
f-circRNA: Fusion circRNA
FISH: Fluorescence in situ hybridization
HSCs: Hematopoietic stem cells
IRES: Internal ribosome entry site
ISS: International Staging System
LFS: Leukemia-free survival
MDS: Myelodysplastic syndromes
miRNA: microRNA
MM: Multiple myeloma
ncRNA: Non-coding RNA
OS: Overall survival
PFS: Progression-free survival
Pre-mRNA: Heterogeneous nuclear RNA
QKI: Quaking protein
qRT-PCR: Quantitative Reverse Transcription-Polymerase Chain Reaction
RBP: RNA-binding protein
RCA reaction: Rolling Circle Amplification reaction
SnRNP: Ribonucleoproteins small nuclear
UTR: Untranslated regionmultiple myelom
